# Supplementary material for: Knowledge integration for physics-informed symbolic regression using pre-trained large language models
Source: Sci Rep. 2026 Jan 13;16:1614. doi: 10.1038/s41598-026-35327-6 (PMC12800073; doi:10.1038/s41598-026-35327-6)
Supplement: Supplementary file 1 — Supplementary Material 1 [file 41598_2026_35327_MOESM1_ESM.docx]

# Appendix

Table [6](#_bookmark138) presents a comparative analysis of the runtime performance between the three SR models (DEAP, PySR, and gplearn) and the three LLMs (Mistral, LLaMA, and Falcon) for each physical experiment (Ball Drop, SHM, and Wave). Namely, the table reports the total runtime of both the SR and LLM components, as well as their relative ratio where the column LLM/SR Ratio quantifies the proportion between the LLM’s total runtime and that of the SR process and the columns Avg SR Per Call and Avg LLM Per Call denote the average time required for a single evaluation of the SR and LLM components, respectively. Similarly, Avg SR Per Iteration and Avg LLM Per Iteration represent the average total runtime per full generation (iteration) of the evolutionary process for the SR and LLM components, respectively. We ran all runtime measurements on a Google Colab Premium environment. The hardware allocation in Colab is somewhat opaque and dynamically assigned. At the beginning of the experiment, we observed CPU and memory configurations to be 32 GB with 2 virtual cores and 100 GB of RAM.

**Table 5: Performance of the LLM-integrated symbolic-regression (SR) model on three physical experiments.**

| **Experiment** | **LLM** | **SR** | **MAE** | **MSE** | **1 − *R*^2^** | **Expression Tree Distance** |
| --- | --- | --- | --- | --- | --- | --- |
|  |  | **DEAP** | **0*.*170** | **0*.*070** | **0*.*18** | **0*.*25** |
|  | **Baseline** | **PySR** | **0*.*150** | **0*.*120** | **0*.*16** | **0*.*19** |
|  |  | **gplearn** | **0*.*180** | **0*.*080** | **0*.*20** | **0*.*10** |
|  |  | **DEAP** | **0*.*120** | **0*.*030** | **0*.*08** | **0*.*05** |
|  | **LLaMA** | **PySR** | **0*.*100** | **0*.*020** | **0*.*06** | **0*.*02** |
| **Dropping ball** |  | **gplearn** | **0*.*150** | **0*.*040** | **0*.*10** | **0*.*12** |
|  |  | **DEAP** | **0*.*140** | **0*.*040** | **0*.*10** | **0*.*10** |
|  | **Falcon** | **PySR** | **0*.*120** | **0*.*030** | **0*.*08** | **0*.*07** |
|  |  | **gplearn** | **0*.*170** | **0*.*050** | **0*.*12** | **0*.*15** |
|  |  | **DEAP** | **0*.*110** | **0*.*030** | **0*.*07** | **0*.*03** |
|  | **Mistral** | **PySR** | **0*.*090** | **0*.*020** | **0*.*05** | **0*.*01** |
|  |  | **gplearn** | **0*.*140** | **0*.*040** | **0*.*09** | **0*.*11** |
|  |  | **DEAP** | **0*.*180** | **0*.*050** | **0*.*12** | **0*.*12** |
|  | **Baseline** | **PySR** | **0*.*170** | **0*.*050** | **0*.*11** | **0*.*13** |
|  |  | **gplearn** | **0*.*180** | **0*.*060** | **0*.*11** | **0*.*18** |
|  |  | **DEAP** | **0*.*080** | **0*.*010** | **0*.*05** | **0*.*02** |
|  | **LLaMA** | **PySR** | **0*.*070** | **0*.*010** | **0*.*04** | **0*.*01** |
| **Simple Harmonic Motion** |  | **gplearn** | **0*.*080** | **0*.*010** | **0*.*05** | **0*.*02** |
|  |  | **DEAP** | **0*.*100** | **0*.*020** | **0*.*07** | **0*.*05** |
|  | **Falcon** | **PySR** | **0*.*090** | **0*.*010** | **0*.*06** | **0*.*03** |
|  |  | **gplearn** | **0*.*120** | **0*.*030** | **0*.*09** | **0*.*12** |
|  |  | **DEAP** | **0*.*070** | **0*.*010** | **0*.*04** | **0*.*01** |
|  | **Mistral** | **PySR** | **0*.*060** | **0*.*010** | **0*.*03** | **0*.*00** |
|  |  | **gplearn** | **0*.*090** | **0*.*020** | **0*.*06** | **0*.*07** |
|  |  | **DEAP** | **0*.*140** | **0*.*090** | **0*.*13** | **0*.*20** |
|  | **Baseline** | **PySR** | **0*.*150** | **0*.*090** | **0*.*12** | **0*.*21** |
|  |  | **gplearn** | **0*.*170** | **0*.*070** | **0*.*15** | **0*.*24** |
|  |  | **DEAP** | **0*.*050** | **0*.*005** | **0*.*03** | **0*.*00** |
|  | **LLaMA** | **PySR** | **0*.*040** | **0*.*004** | **0*.*02** | **0*.*00** |
| **Electromagnetic Wave** |  | **gplearn** | **0*.*070** | **0*.*010** | **0*.*05** | **0*.*05** |
|  |  | **DEAP** | **0*.*070** | **0*.*010** | **0*.*05** | **0*.*02** |
|  | **Falcon** | **PySR** | **0*.*060** | **0*.*008** | **0*.*04** | **0*.*01** |
|  |  | **gplearn** | **0*.*090** | **0*.*020** | **0*.*07** | **0*.*10** |
|  |  | **DEAP** | **0*.*040** | **0*.*004** | **0*.*02** | **0*.*00** |
|  | **Mistral** | **PySR** | **0*.*030** | **0*.*003** | **0*.*01** | **0*.*00** |
|  |  | **gplearn** | **0*.*060** | **0*.*009** | **0*.*04** | **0*.*04** |

**Table 6: Comparison of SR and LLM runtimes across experiments.**

| **Experiment** | **SR Tool** | **LLM** | **Total SR (s)** | **Total LLM (s)** | **LLM/SR Ratio** | **Avg SR / Call** | **Avg LLM / Call** | **Avg SR / Iter.** | **Avg LLM / Iter.** |
| --- | --- | --- | --- | --- | --- | --- | --- | --- | --- |
|  | **DEAP** | **Mistral** | **7.8** | **55.1** | **7.1** | **0.3** | **2.3** | **2.6** | **18.4** |
|  | **DEAP** | **LLaMA** | **7.3** | **141.3** | **19.4** | **0.3** | **5.9** | **2.4** | **47.1** |
|  | **DEAP** | **Falcon** | **7.4** | **49.3** | **0.0** | **0.3** | **0.0** | **2.5** | **0.1** |
| **Ball Drop** | **PySR** | **Mistral** | **169.9** | **56.6** | **0.3** | **7.1** | **2.4** | **56.6** | **18.9** |
|  | **PySR** | **LLaMA** | **151.4** | **58.5** | **0.4** | **6.3** | **2.4** | **50.5** | **19.5** |
|  | **PySR** | **Falcon** | **154.9** | **47.8** | **0.0** | **6.5** | **0.0** | **51.6** | **0.1** |
|  | **gplearn** | **Mistral** | **1.7** | **54.0** | **31.0** | **0.1** | **2.3** | **0.6** | **18.0** |
|  | **gplearn** | **LLaMA** | **1.7** | **134.5** | **76.9** | **0.1** | **5.6** | **0.6** | **44.8** |
|  | **gplearn** | **Falcon** | **1.7** | **36.1** | **0.1** | **0.1** | **0.0** | **0.6** | **0.1** |
|  | **DEAP** | **Mistral** | **7.2** | **54.2** | **7.5** | **0.3** | **2.3** | **2.4** | **18.1** |
|  | **DEAP** | **LLaMA** | **7.6** | **140.4** | **18.5** | **0.3** | **5.9** | **2.5** | **46.8** |
|  | **DEAP** | **Falcon** | **7.2** | **38.2** | **0.0** | **0.3** | **0.0** | **2.4** | **0.1** |
| **SHM** | **PySR** | **Mistral** | **183.0** | **56.8** | **0.3** | **7.6** | **2.4** | **61.0** | **18.9** |
|  | **PySR** | **LLaMA** | **191.0** | **67.6** | **0.4** | **8.0** | **2.8** | **63.7** | **22.5** |
|  | **PySR** | **Falcon** | **186.2** | **41.9** | **0.0** | **7.8** | **0.0** | **62.1** | **0.1** |
|  | **gplearn** | **Mistral** | **1.7** | **43.1** | **24.9** | **0.1** | **1.8** | **0.6** | **14.4** |
|  | **gplearn** | **LLaMA** | **1.7** | **109.2** | **62.9** | **0.1** | **4.5** | **0.6** | **36.4** |
|  | **gplearn** | **Falcon** | **1.7** | **26.0** | **0.1** | **0.1** | **0.0** | **0.6** | **0.1** |
|  | **DEAP** | **Mistral** | **7.3** | **48.5** | **6.6** | **0.3** | **2.0** | **2.4** | **16.2** |
|  | **DEAP** | **LLaMA** | **7.2** | **72.9** | **10.1** | **0.3** | **3.0** | **2.4** | **24.3** |
|  | **DEAP** | **Falcon** | **7.3** | **35.6** | **0.0** | **0.3** | **0.0** | **2.4** | **0.1** |
| **Wave** | **PySR** | **Mistral** | **173.0** | **55.1** | **0.3** | **7.2** | **2.3** | **57.7** | **18.4** |
|  | **PySR** | **LLaMA** | **167.8** | **60.8** | **0.4** | **7.0** | **2.5** | **55.9** | **20.3** |
|  | **PySR** | **Falcon** | **169.9** | **30.7** | **0.0** | **7.1** | **0.0** | **56.6** | **0.1** |
|  | **gplearn** | **Mistral** | **1.8** | **53.5** | **30.4** | **0.1** | **2.2** | **0.6** | **17.8** |
|  | **gplearn** | **LLaMA** | **1.7** | **82.5** | **47.5** | **0.1** | **3.4** | **0.6** | **27.5** |
|  | **gplearn** | **Falcon** | **1.7** | **39.5** | **0.1** | **0.1** | **0.0** | **0.6** | **0.1** |
